# Milk Inspection in New York

**Published:** 1900-03-31

**Authors:** 


### MILK INSPECTION IN NEW YORK.

So evident is the part borne by milk in the causation and distribution of disease that a growing willingness is showing itself in this country to extend the restrictions imposed by law upon free trade in this useful but dangerous article of diet. In New York, where, when once they make up their minds to a thing they are apt to do it thoroughly, the trade in milk is brought very completely under the control of the municipal authorities.

From a paper by Dr. Herman Betz in the New York *Medical News*, we gather that since the year 1896 the

Department of Health has required every dealer in milk to take out a permit, to obtain which he must apply in person, and fill up a form, stating, among other things, the kind of business he is engaged in, the amount of milk, approximately, which he expects to sell per day, the dealer from whom he is to derive his supply, the hour of the day the delivery takes place, the marks to be found on his cans, and also whether he intends to handle any condensed milk in bulk. The proposed place of business is afterwards inspected, and if all is found right a permit is issued without charge; but it is revocable at the pleasure of the Board of Health, and must always be displayed in a conspicuous place. Should a dealer be twice convicted of selling adulterated milk, the permit is taken from him and he is never allowed to sell milk again. The wholesale dealer must give the name and address of the farmer from whom the milk is received, the station from which it is shipped, the railroad carrying it, the time at which the milk arrives at the terminal station, and the number of hours occupied in the transit, together with a number of details concerning the farm and its water supply and dairy arrangements. To the wholesale dealer not only is a store permit necessary, but wagon permits, one for each wagon he employs. He must also give the name and address of each driver, and the number of the permit which the driver carries must be painted on the side of the wagon which he drives. Thus it is possible to check the statements of the porters and drivers at any point, and the introduction of unlicensed milk is prevented. Lastly, arrangements are made between the Health Department of New York and those of the various States from which the milk is shipped for the latter to notify at once the occurrence of any contagious or infectious disease at any place within their jurisdiction from which milk is shipped, and such notifications are forwarded by telegraph. So as to render the information so received immediately available for purposes of disease-prevention, a record is kept of the various railways and the districts they tap, of the parties in New York who receive milk from such places, and of the amounts they are in the habit of taking. Thus not only is there a very complete system for the inspection of all milk which is exposed or offered for sale, but the milk can be traced in transit, and wholesale distribution of milk is brought under such control that a great check is placed upon that habit of mixing milk which makes it so difficult and sometimes impossible to trace an epidemic to its source. It is to be noted that the milk inspectors are men possessed of a certain amount of scientific training, and that the milk analysis is performed in the State laboratory in a very systematic manner, so that it is now very rare for the official decision to be called in question. Nothing is said about the cost of this system of inspection, but it must be considerable. Good work, however, can never be cheap.

